# Formal Consensus Method to Evaluate the Conformity of Prescription of a Recently Approved Chemotherapy Treatment in an Observatory Study

**DOI:** 10.1371/journal.pone.0123035

**Published:** 2015-04-02

**Authors:** Nadine Houédé, Eric Leutenegger, Mariella Lomma, Carine Bellera

**Affiliations:** 1 Department of Medical Oncology, Nîmes University Hospital, Place du Pr R. Debré 30000, Nîmes, France; 2 INSERM U1194, Institut de Recherche en Cancérologie de Montpellier, Montpellier, France; 3 Late Phase Studies, Gecem, Paris, France; 4 Department of Clinical Epidemiology, Nîmes University Hospital, Place du Pr R. Debré 30000, Nîmes, France; 5 Clinical Research and Clinical Epidemiology Unit, Institut Bergonié, Comprehensive Cancer Center, Bordeaux, France; 6 INSERM CIC 1401, Clinical Epidemiology, Bordeaux, France; Texas Tech University Health Sciences Center, UNITED STATES

## Abstract

Cabazitaxel is a second line chemotherapy drug recently approved for the treatment of metastatic castration-resistant prostate cancer. A first panel of French experts and a second independent panel of European experts were convened to assess the conformity of prescription of cabazitaxel with a Delphi consensus method. A two-round modified Delphi consensus process was conducted. This methodology is based on experts’ opinion obtained in a systematic manner. The process was divided into five steps: (i) elaboration of the questionnaire, (ii) rating, (iii) analysis, (iv) discussion of the points with absence of consensus following rating of the questionnaire, and (v) final reporting. Consensus was defined according to RAND method and all analyses were conducted according to the same methodology. At the end of the two rounds of rating and a synthesis meeting, of the 26 items included in the Summary of Product Characteristics (SPC), 11 items were judged appropriate with strong consensus by the two independent panels of experts. These items can therefore be considered of prime importance to evaluate conformity of cabazitaxel prescription in the context of observatory studies as well as in further clinical trials using this new taxane. Our findings further provide important evidence about the value of the Delphi consensus and highlight a requirement for “conformity” standards to assist practitioners in a safe chemotherapy drug prescription.

## Introduction

Prostate cancer is the most commonly diagnosed cancer in men aged over 50 and it is the fifth leading cause of death from cancer in men worldwide, with an estimation of over a million of new cases and 307,000 deaths in 2012 (http://globocan.iarc.fr; accessed December 2013).

For metastatic castration-resistant prostate cancer (mCRPC) patients, treatment usually consists in a first line chemotherapy with docetaxel followed by a second line treatment with either second generation hormonal therapies (abiraterone acetate or enzalutamide) or the new taxane derivative cabazitaxel.

Cabazitaxel (Jevtana) is a semi-synthetic taxane with a mechanism of action different to that of docetaxel and paclitaxel. It is an antineoplastic agent that stabilises the microtubules network via its binding to tubulins and the inhibition of microtubules disassembly. Cabazitaxel has demonstrated a broad spectrum of antitumour activity against advanced human tumours xenografted in mice. It is active in docetaxel-sensitive tumours and in tumour models resistant to chemotherapy, including docetaxel. The efficacy of cabazitaxel has been demonstrated in the pivotal clinical trial TROPIC, a randomized, open-label, phase III study that compared cabazitaxel (n = 378) to mitoxantrone (n = 377), both associated with prednisone or prednisolone in patients with hormone-refractory metastatic prostate cancer previously treated with a docetaxel containing regimen [1]. The median overall survival (OS) was significantly increased in the cabazitaxel-treated group compared to the mitoxantrone-treated group (15.1 *versus* 12.7 months respectively P<0.0001) independent of the duration of androgen deprivation therapy [1]. Most common adverse event that can lead to a treatment discontinuation are haematological (neutropenia 82%), gastrointestinal (diarrhoea 6%) and general disorders (asthenia 5%). A recent study analysed the data of the TROPIC study on efficacy and toxicity observed in the subgroup of patients included in French centres. Among the 90 patients enrolled in France, the median OS was 18 months for the cabazitaxel arm *versus* 14.3 months for the mitoxantrone arm. Also, the most common grade ≥3 adverse events were hematologic with neutropenia (89%), most commonly afebrile (6.5%) and digestive with 4% of patients reporting diarrhoea. These results are comparable to those reported for the overall population and the safety profile remains favourable without any toxic death related to cabazitaxel [2].

Following TROPIC trial, cabazitaxel (in combination with prednisone or prednisolone) was approved by the U.S. Food and Drug Administration (FDA) on June 2010 and by the European Medicines Agency (EMA) on March 2011 for the treatment of hormone-refractory prostate cancer. In France cabazitaxel was approved in 2012 but made available only in 2014.

To investigate whether the precautions for the use of cabazitaxel (contraindications, hepatic function, premedication for hypersensitivity, prophylactic treatments, dose and dose adjustment, as well as conditions of administration) are respected in routine practice, an observational cohort study will be implemented in 32 oncology centres of South-West region of France (CABOBS study). The primary objective of this cohort is to evaluate the conformity of cabazitaxel prescriptions with the Summary of Product Characteristics (SPC) [3] in clinical practice (indications and monitoring methods) and according to the conditions of use.

Based on the SPC, Specifications for prescription include 26 items divided into “baseline” (14) criteria and “cycle 2” (12) criteria, which include requirements for the second and all following cycles. Because of such high number of specific items, strict adherence to prescription indications might be unsatisfactory or not appropriate from the clinical point of view, each criterion has a different relevance on the appropriateness of prescription; therefore, it is necessary to select the criteria based on their relevance and according to the opinion of the clinical experts.

For this purpose, two independent panels of experts in the field of uro-oncology, one French panel and a European panel from EORTC, were solicited in order to define the importance of each criterion of the SPC and to allow a qualitative assessment of conformity using a formal consensus method.

Consensus methods provide means of synthesising information and compare contradictory opinions or evidences on a specific issue. Their purpose is to assess the extent of agreement and to resolve disagreement among a group of selected individuals by identifying and selecting, through iterative ratings with feedback, specific points on which there is disagreement or uncertainty. The Delphi method, a consensus method which originated in 1948, is an attempt to obtain expert opinion in a systematic manner [4, 5]. The survey is conducted over “rounds” in which questionnaires are administered to the experts individually and anonymously. After each round, the results are listed and reported to the group. A Delphi is considered complete when there is a convergence of opinion. In a modified Delphi usually a predefined number of rounds and a final round, in which experts meet to resolve and summarise the results of the consensus, are held.

We relied on the Delphi method as it is most suited to areas where a limited number of evidences are available to enable a recommendation or guidelines to be drawn. Moreover, all the evaluated items came from an official document provided by the EMA, the SPC of cabazitaxel [3]. Thus, it is expected from the experts to select the most relevant items.

In the frame of the CABOBS study, this method will allow to select each of the 26 items of the SPC list according to their importance, and to define the primary objective of conformity to cabazitaxel conditions of use.

## Methods

### Study design

**Fig 1:**
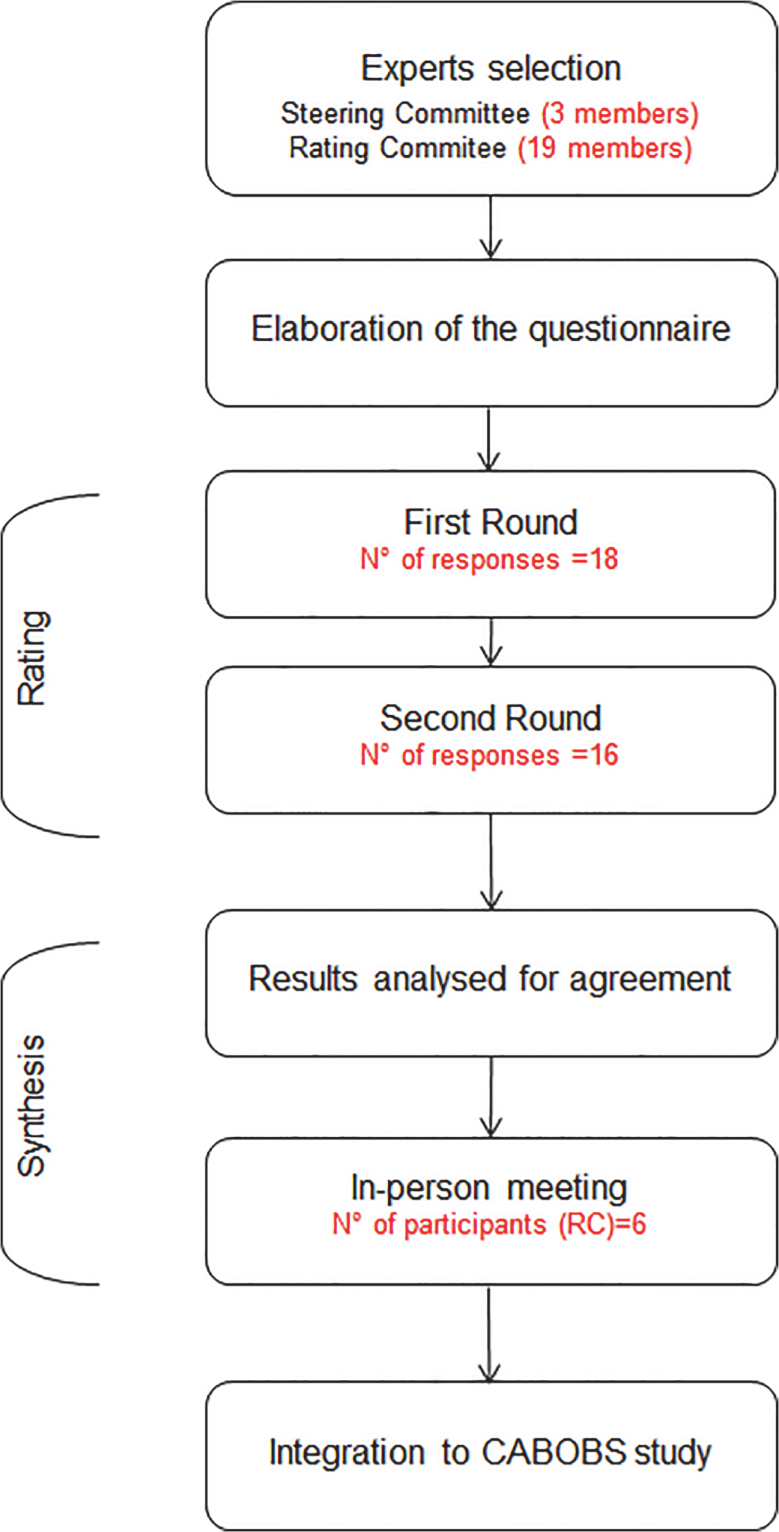
Flowchart.

Formal consensus process was developed following a modified Delphi consensus method with the rating process based on the RAND/UCLA scoring methodology [6, 7]. The process described in Fig 1 was divided into the following steps: (i) elaboration of the questionnaire, (ii) rating, (iii) analysis,(iv) discussion of the points without absence of the consensus following rating of the questionnaire and (v) final reporting. No ethical approval was required to perform this study.

### Experts’ committees

Three independent committees were involved in the study: the steering committee and two independent rating committees. The steering committee was composed of an oncologist (NH), a biostatistician (CB) and an epidemiologist from an independent contract research organization (EL). These members were initially implicated in the design and the promotion of the observational study. The steering committee was involved in all steps except in the rating process.

The two independent rating committees involved in the rating process were composed of experts in uro-oncology who were solicited within the French GETUG’s group (Groupe d’Etude des Tumeurs Uro-Génitales) for the first panel of experts, and within the EORTC GU group for the second panel of experts. The nominative lists of experts who accepted to participate to these rating committees are provided in the acknowledgments section.

### Elaboration of the questionnaire

The elaboration of the questionnaire was carried out by the steering committee. All items listed in the SPC were included in the questionnaire and separated into “baseline” and “cycle 2” items. It was assumed that if prescriptions of the first and second cycles were in compliance with SPC, practitioner prescription could be considered as conform.

### Rating and analysis of the questionnaires

In this phase, which takes place in three steps, the items on which the members of the rating group agree and those on which they differ or are undecided were identified by means of a vote conducted in two rounds. Initially, the first questionnaire was sent by email to each member of the rating group for individual rating. The rules for the rating and the analysis of the scores were defined *a priori* and communicated to the rating group, prior to the first round.

Step1: for each item, rating experts were asked to indicate on a scale ranging from one (totally disagree) to nine (totally agree) how the specific item was relevant for cabazitaxel prescription. An item was defined as appropriate, i.e. to be satisfied to consider the prescription to be conform, if the median of all scores was ≥7 and there was agreement among all members of the rating committee (range of rating 7–9 for strong consensus and 5–9 for relative consensus); inappropriate, i.e. this item did not need to be satisfied to consider the prescription to be conform, if the median of all scores was ≤3.5 and there was agreement among all members of the RC (range of rating 1–3 for strong consensus and 1–5 for relative consensus); uncertain if the median of all scores was comprised between 4 and 6.5 or in case of absence of consensus (S1 Table).

Step 2: at the end of the first round, only items with a strong consensus were retained; all other items were inserted in the second questionnaire for the second round of rating.

Therefore, the second questionnaire was drafted including only the items with absence of strong consensus (see next paragraph) and was sent to each member of the rating group along with the results of the first round and a copy of their first round ratings. Each expert was asked to rate the new questionnaire with the same scale used for the first round. The same rating methodology as for the first round was applied, but rules were slightly modified according to the number of participants. For a rating committee with more than 16 (N≥16) two “abnormal” (outlier) or missing scores could be accepted. Consequently, the definition of strong or relative consensus on the appropriateness of an item was modified accordingly (S2 Table).

Step 3: in-person meeting. After analysis of the second round of rating, a face-to-face and a conference call meetings were organised with both steering and ratings groups’ members in order to confirm items on which there was an agreement and discuss those on which the consensus was missing. The meetings were led by a representative of the steering committee. All members of the steering and the ratings committees were allowed to attend, however only rating experts were asked to provide with their feedback. Prior to the meetings, an agenda was sent to the experts, as well as supporting documents. These documents included a brief reminder of the objectives of the project, the decision rules to define the presence or the absence of consensus, and a summary report of the analysis of the second round including items to be discussed during the meeting.

### Final reporting

The in-person meetings led to a final proposition that represented the results of the formal consensus and was sent to all the experts. Subsequently, after approval of the rating committee, the list of items with their relative conformity ratings was distributed to all investigators of the centres participating to the CABOBS study.

## Results

### Elaboration of the questionnaire and selection of the two rating committees

The first questionnaire was elaborated according to the principal objective of the CABOBS observational study which sought to evaluate the conformity of cabazitaxel prescriptions with the SPC for the treatment of mCRPC.

All items listed in the SPC were included in the questionnaire and separated into “baseline” and “cycle 2” items. The questionnaire sent for rating for the first round is shown in Table 1.

**Table 1:** Results of the rating process following the first round for the first panel of French experts.

				N° of responses									
	Criterion	N	Median	1	2	3	4	5	6	7	8	9	C
Baseline	1) Histologically proven prostate adenocarcinoma	18	9.0								2	16	**S**
	2) Chemical (on-going) or surgical castration	18	9.0								1	17	**S**
	3) Metastatic cancer	18	9.0									18	**S**
	4) Previously treated with docetaxel	18	9.0						1		1	16	**R**
	5) Performans status < 2	18	7.0						6	5	3	4	**R**
	6) Clinical examination	18	9.0							4	2	12	**S**
	7) Biological test (complete blood count, haemostasis)	18	9.0							1		17	**S**
	8) Leucocytes > 3000 or neutrophiles >1500mm^3^	18	8.0						3	3	4	8	**R**
	9) Platelets > 100000/mm^3^	18	8.5					1	2	2	4	9	**R**
	10) ALT and/or AST<1.5x ULN or bilirubin<1 ULN	18	7.0					2	4	6	4	2	**R**
	11) Prophylactic treatment (antihistamine, H2-antagonist, corticosteroids)	18	8.0					1	4	4		9	**R**
	12) Maximal dose 25mg/m^2^	18	9.0						1			17	**R**
	13) IV infusion over 1 hour (± 1h)	18	8.5						5	3	1	9	**R**
	14) Concomitant corticoids prescription (prednisone or prednisolone 10mg/day)	18	6.0				2	3	5	2	2	4	**N**
Cycle 2	1) Intercycle between 3 and 5 weeks	18	8.0					1		5	6	6	**R**
	2) Performans status < 2	18	8.0						5	2	6	5	**R**
	3) Clinical examination	18	9.0						3	2	3	10	**R**
	4) Biological test (complete blood count, haemostasis)	18	9.0							1		17	**S**
	5) Leucocytes > 3000 or neutrophiles > 1500mm^3^	18	9.0					1		5	2	10	**R**
	6) Platelets > 100000/mm^3^	18	9.0				1			4	3	10	**N**
	7) ALT and/or AST<1.5x ULN or bilirubin<1 ULN	18	7.0					2	1	8	4	3	**R**
	8) Prophylactic treatment (antihistamine, H2-antagonist, corticosteroids)	18	9.0						2	6		10	**R**
	9) Maximal dose 25mg/m^2^	18	9.0						1	2		15	**R**
	10) IV infusion over 1 hour (± 1h)	18	7.5					1	5	3	1	8	**R**
	11) Concomitant corticoids prescription (prednisone or prednisolone 10mg/day)	18	7.0				2	2	4	4	1	5	**N**
	12) Dose adjustment (25mg/m^2^ to 20mg/m^2^) in case of neutropenia, diarrhoea followed by hospitalisation, peripheral neuropathy	18	9.0						1	1	3	13	**R**

Experts are asked to rate each item according to its relevance for prescription (Abbreviations: C, consensus; S, strong; R, Relative; N, absence of consensus; AST, aspartate aminotransferase; ALT, alanine aminotransferase; ULN, upper limit of normal; IV, intravenous infusion).

Two independent panels of experts in onco-urology were solicited: a first panel of French experts who belonged to the GETUG (Groupe d’Etude des Tumeurs Uro-génitales), a French national uro-oncology network, were invited to participate. Among the 40 solicited experts, 18 accepted to participate and received the questionnaires; a second independent panel of European experts who belonged to the EORTC GU (European Organization for Research and Treatment of Cancer Genito-Urinary) group. Among the 57 solicited medical oncologist experts, 16 accepted to participate to the study but only 15 experts provided answers to the second round questionnaire. The nominative list of experts for each panel is detailed in the acknowledgments section.

### First round

For the French panel, the analysis of the first round of rating showed that six items (baseline items 1, 2, 3, 6, 7; and cycle 2 item 4) had a median score ≥7 with a strong consensus (score range from 8 to 9) and therefore were judged appropriate; other 17 items were judged appropriate with a median score ≥7 and relative consensus (score range from 5 to 9) (baseline items 4, 5, 8, 9, 10, 11, 12, 13; and cycle 2 items 1, 2, 3, 5, 7, 8, 9, 10, 12); finally three items there were judged uncertain, two of which with no consensus (cycle 2 items 6 and 11: median ≥7 and a score range from 4 to 9) and one with indecision (baseline item 14: median = 6 and a score range from 4 to 9) (Table 1). Therefore, excluding the six “strong consensus” items all the others were listed in the second questionnaire and subjected to a new rating. Each expert was provided again with a rating scale (same principle as for the first round), its previous round rating and the median score obtained at the first round for each item (Table 1). Therefore, during the first round a strong consensus was obtained for 7 items (27%), relative consensus for 16 items (61%) and uncertainty or indecision for 3 items (11%). As a result, a total of 19 items were subject to rating in the second round.

For the European panel, results of the first round showed that all items had a median score ≥7 with a strong consensus for 5 items (baseline items 3, 7 and cycle 2 items 4, 9, 12), a relative consensus for 8 items (baseline items 4, 5, 6, 8, 9 and cycle 2 items 5, 6, 11), and 13 items were judged uncertain. A total of 21 items were subject to rating in the second round (Table 2).

**Table 2:** Results of the rating process following the first round for the second panel of European experts.

				N° of responses									
	Criterion	N	Median	1	2	3	4	5	6	7	8	9	C
Baseline	1) Histologically proven prostate adenocarcinoma	16	9.0	1	1			1		2	2	9	**N**
	2) Chemical (on-going) or surgical castration	16	9.0			1	1			2	3	9	**N**
	3) Metastatic cancer	16	9.0								2	14	**S**
	4) Previously treated with docetaxel	16	9.0					2	1	1	2	10	**R**
	5) Performans status < 2	15	7.5					2	1	4	3	5	**R**
	6) Clinical examination	16	8.0					2	1	1	4	8	**R**
	7) Biological test (complete blood count, haemostasis)	16	9.0							1	2	13	**S**
	8) Leucocytes > 3000 or neutrophiles >1500mm^3^	16	8.5					1		2	5	8	**R**
	9) Platelets > 100000/mm^3^	16	8.5					2		1	6	7	**R**
	10)ALT and/or AST<1.5x ULN or bilirubin<1 ULN	16	7.0			1	2	1	3	6	2	1	**N**
	11) Prophylactic treatment (antihistamine, H2-antagonist, corticosteroids)	16	9.0		1				1	1	4	9	**N**
	12) Maximal dose 25mg/m^2^	16	9.0		1				2	1	3	9	**N**
	13) IV infusion over 1 hour (± 1h)	16	7.0		1	1		2	3	1	3	5	**N**
	14) Concomitant corticoids prescription (prednisone or prednisolone 10mg/day)	16	7.0	1	1		2	1	2	3	2	4	**N**
Cycle 2	1) Intercycle between 3 and 5 weeks	16	7.0	1		1	2	1	1	3	2	5	**N**
	2) Performans status < 2	16	7.0	1			1	1	2	6	2	3	**N**
	3) Clinical examination	16	7.5	1		1	1	1	2	2	2	6	**N**
	4)Biological test (complete blood count, haemostasis)	15	9.0							2	1	12	**S**
	5) Leucocytes > 3000 or neutrophiles >1500mm^3^	15	9.0					1		3	3	8	**R**
	6) Platelets > 100000/mm^3^	16	8.0					1	1	2	5	7	**R**
	7) ALT and/or AST<1.5x ULN or bilirubin<1 ULN	16	7.0			1	1	1	4	3	4	2	**N**
	8) Prophylactic treatment (antihistamine, H2-antagonist, corticosteroids)	16	8.5		1					4	3	8	**N**
	9) Maximal dose 25mg/m^2^	16	8.5							4	4	8	**S**
	10) IV infusion over 1 hour (± 1h)	15	7.0		1	1		1	3	2	3	4	**N**
	11) Concomitant corticoids prescription (prednisone or prednisolone 10mg/day)	15	7.5					3	2	2	4	4	**R**
	12) Dose adjustment (25mg/m^2^ to 20mg/m^2^) in case of neutropenia, diarrhoea followed by hospitalisation, peripheral neuropathy	16	9.0							2	4	10	**S**

Experts are asked to rate each item according to its relevance for prescription (Abbreviations: C, consensus; S, strong; R, Relative; N, absence of consensus; AST, aspartate aminotransferase; ALT, alanine aminotransferase; ULN, upper limit of normal; IV, intravenous infusion).

### Second round

For the French panel, the results of the second round of rating showed that six new items were judged appropriate with a median score ≥7 and a score range from 7 to 9 (baseline items 9, 11, 12; and cycle 2 items, 2, 8, 9). Three items were judged uncertain: for two the median score was ≥7 but with a score range from 4 to 9, meaning an absence of consensus (baseline items 8 and 14); and one with median score = 6.5 and a score range from 1 to 9 (cycle 2 item 11) (Table 3). At the end of the second round, a strong consensus was obtained for 12 items (46%), relative consensus for 11 items (42%) and uncertainty or indecision for 3 items (11%).

**Table 3:** Results of the rating process following the second round for the first panel of French experts.

				N° of responses									
	Criterion	N	Median	1	2	3	4	5	6	7	8	9	C
Baseline	1) Histologically proven prostate adenocarcinoma	18	9.0	No second rating									**S**
	2) Chemical (on-going) or surgical castration	18	9.0	No second rating									**S**
	3) Metastatic cancer	18	9.0	No second rating									**S**
	4) Previously treated with docetaxel	16	9.0					1				15	**R**
	5) Performans status < 2	16	7.0					2	2	8	3	1	**R**
	6) Clinical examination	18	9.0	No second rating									**S**
	7)Biological test (complete blood count, haemostasis)	18	9.0	No second rating									**S**
	8) Leucocytes > 3000 or neutrophiles >1500mm^3^	16	8.0				1	1		4	3	7	**N**
	9) Platelets > 100000/mm^3^	16	9.0							5	2	9	**S**
	10) ALT and/or AST<1.5x ULN or bilirubin<1 ULN	16	7.0					1	2	7	4	2	**R**
	11) Prophylactic treatment (antihistamine, H2-antagonist, corticosteroids)	16	9.0							1	4	11	**S**
	12) Maximal dose 25mg/m^2^	16	9.0							2		14	**S**
	13) IV infusion over 1 hour (± 1h)	16	8.0					3		5		8	**R**
	14) Concomitant corticoids prescription (prednisone or prednisolone 10mg/day)	16	7.0	1			1	1	4	5		4	**N**
Cycle 2	1) Intercycle between 3 and 5 weeks	16	8.5					1		3	4	8	**R**
	2) Performans status < 2	16	8.0							6	8	2	**S**
	3) Clinical examination	16	9.0					2		1	3	10	**R**
	4) Biological test (complete blood count, haemostasis)	18	9.0	No second rating									**S**
	5) Leucocytes > 3000 or neutrophiles >1500mm^3^	15	8.0					1		4	3	7	**R**
	6) Platelets > 100000/mm^3^	16	9.0					1		4	2	9	**R**
	7) ALT and/or AST<1.5x ULN or bilirubin<1 ULN	16	7.0					1	2	8	3	2	**R**
	8) Prophylactic treatment (antihistamine, H2-antagonist, corticosteroids)	16	9.0							2	2	12	**S**
	9) Maximal dose 25mg/m^2^	16	9.0							2		14	**S**
	10) IV infusion over 1 hour (± 1h)	15	7.0					1	2	5	1	6	**R**
	11) Concomitant corticoids prescription (prednisone or prednisolone 10mg/day)	16	6.5	1			1	1	5	3	1	4	**N**
	12) Dose adjustment (25mg/m^2^ to 20mg/m^2^) in case of neutropenia, diarrhoea followed by hospitalisation, peripheral neuropathy	16	9.0					1		1	2	12	**R**

Experts are asked to rate each item according to its relevance for prescription in light of the responses to the first round questionnaire (Abbreviations: C, consensus; S, strong; R, Relative; N, absence of consensus; AST, aspartate aminotransferase; ALT, alanine aminotransferase; ULN, upper limit of normal; IV, intravenous infusion).

For the European panel, the results of the second round of rating showed five new items judged appropriate with a median score ≥7 and a score range from 7 to 9 (baseline items 4, 12; and cycle 2 items, 2, 5, 6). Two items were judged uncertain with a median score ≥7 but with a score range from 2 to 9, meaning an absence of consensus (baseline items 14 and cycle 2 item 3). According to the Delphi methodology, one outlier was removed for each item with weak consensus or absence of consensus, leading to the validation of 8 more items with strong consensus (baseline items 2, 5, 6, 8, 9, 11; cycle 2 items 7, 8) and 3 items with weak consensus (baseline items 1; cycle 2 items 10, 11). In fine, a strong consensus was obtained for 18 items (69%), relative consensus for 6 items (23%), and uncertainty or indecision for 2 items (8%) (Table 4).

**Table 4:** Results of the rating process following the second round for the second panel of European experts.

				N° of responses									
	Criterion	N	Median	1	2	3	4	5	6	7	8	9	C
Baseline	1) Histologically proven prostate adenocarcinoma	15	8.5		1			1		1	4	8	R
	2) Chemical (on-going) or surgical castration	15	9.0			1					1	13	S
	3) Metastatic cancer	15		No second rating									S
	4) Previously treated with docetaxel	15	9.0						2	1	1	11	S
	5) Performans status < 2	15	8.0					1		2	7	5	S
	6) Clinical examination	15	8.5					1		2	6	6	S
	7)Biological test (complete blood count, haemostasis)	15		No second rating									**S**
	8) Leucocytes > 3000 or neutrophiles >1500mm^3^	15	8.0					1		2	7	5	S
	9) Platelets > 100000/mm^3^	15	8.0						2	1	7	5	S
	10)ALT and/or AST<1.5x ULN or bilirubin<1 ULN	15	7.0				1	1	1	7	4	1	R
	11) Prophylactic treatment (antihistamine, H2-antagonist, corticosteroids)	15	9.0		1						5	9	S
	12) Maximal dose 25mg/m^2^	15	9.0							1	2	12	S
	13) IV infusion over 1 hour (± 1h)	15	7.5				1	1	1	4	3	5	R
	14) Concomitant corticoids prescription (prednisone or prednisolone 10mg/day)	15	7.5		1		1	1	1	5	2	4	N
Cycle 2	1) Intercycle between 3 and 5 weeks	15	7.0				1	1		10	1	2	R
	2) Performans status < 2	15	8.0							4	6	4	S
	3) Clinical examination	15	7.5			1	1	1	1	4	3	4	N
	4) Biological test (complete blood count, haemostasis)	15		No second rating									**S**
	5) Leucocytes > 3000 or neutrophiles >1500mm^3^	15	8.5							3	5	7	S
	6) Platelets > 100000/mm^3^	15	8.0							3	7	5	S
	7) ALT and/or AST<1.5x ULN or bilirubin<1 ULN	15	8.0				1			2	9	3	S
	8) Prophylactic treatment (antihistamine, H2-antagonist, corticosteroids)	15	8.5			1					6	8	S
	9) Maximal dose 25mg/m^2^	15		No second rating									**S**
	10) IV infusion over 1 hour (± 1h)	15	7.5				1	1	1	5	1	6	R
	11) Concomitant corticoids prescription (prednisone or prednisolone 10mg/day)	15	7.0		1			2		6	2	4	R
	12) Dose adjustment (25mg/m^2^ to 20mg/m^2^) in case of neutropenia, diarrhoea followed by hospitalisation, peripheral neuropathy	15		No second rating									**S**

Experts are asked to rate each item according to its relevance for prescription in light of the responses to the first round questionnaire (Abbreviations: C, consensus; S, strong; R, Relative; N, absence of consensus; AST, aspartate aminotransferase; ALT, alanine aminotransferase; ULN, upper limit of normal; IV, intravenous infusion).

### In-person meeting

For the French panel, the final consensus meeting took place in Paris on the 5^th^ of February 2014 at the national cancer institution headquarters. Six members of the rating group and the steering committee were present.

Only the 14 items for which no strong consensus was reached after the second round were discussed. During the meeting the experts of the rating committee, because of the presence of only one outlier, suggested the consensus to be changed for three items from relative to strong; therefore they were judged “appropriate”(baseline items 4, cycle2items 1 and 12). For one item, on which there was an absence of consensus, one outlier score was excluded and consequently the item was defined as “appropriate” with a relative consensus (baseline item 8). The relative consensus was confirmed for seven items that were defined “appropriate” (baseline items 5, 10, 13 and cycle 2 items 3, 5, 7 and 10). Finally, the absence of consensus was confirmed for two items: hence, they were defined “inappropriate” (baseline 14 and cycle 2 item 11). In summary, after the two rounds of rating and the in-person meeting, 16 items out of 26 were judged “appropriate” with a strong consensus, eight “appropriate” with a relative consensus and two “inappropriate” (Table 5).

**Table 5:** Final results of the rating process.

	Criterion	French panel	EORTC panel
Baseline	1) Histologically proven prostate adenocarcinoma	S	R
	2) Chemical (on-going) or surgical castration	S	S
	3) Metastatic cancer	S	S
	4) Previously treated with docetaxel	S	S
	5) Performance status < 2	R	S
	6) Clinical examination	S	S
	7)Biological test (complete blood count, haemostasis)	S	S
	8) Leucocytes > 3000 or neutrophils >1500mm^3^	R	S
	9) Platelets > 100000/mm^3^	S	S
	10)ALT and/or AST<1.5x ULN or bilirubin<1 ULN	R	R
	11) Prophylactic treatment (antihistamine, H2-antagonist, corticosteroids)	S	S
	12) Maximal dose 25mg/m^2^	S	S
	13) IV infusion over 1 hour (± 1h)	R	R
	14) Concomitant corticoids prescription (prednisone or prednisolone 10mg/day)	NC	R
Cycle 2	1)Intercycle between 3 and 5 weeks	S	R
	2) Performance status < 2	S	S
	3) Clinical examination	R	R
	4)Biological test (complete blood count, haemostasis)	S	S
	5) Leucocytes > 3000 or neutrophils >1500mm^3^	R	S
	6) Platelets > 100000/mm^3^	S	S
	7) ALT and/or AST<1.5x ULN or bilirubin<1 ULN	R	S
	8) Prophylactic treatment (antihistamine, H2-antagonist, corticosteroids)	S	S
	9) Maximal dose 25mg/m^2^	S	S
	10)IV infusion over 1 hour (± 1h)	R	R
	11) Concomitant corticoids prescription (prednisone or prednisolone 10mg/day)	NC	R
	12) Dose adjustment (25mg/m^2^ to 20mg/m^2^) in case of neutropenia, diarrhoea followed by hospitalisation, peripheral neuropathy	S	S

The results are grouped into: Mandatory for conformity; Relative consensus; No consensus (Abbreviations: AST, aspartate aminotransferase; ALT, alanine aminotransferase; ULN, upper limit of normal; IV, intravenous infusion).

For the European panel, the in-person meeting was a conference call which took place on February 9^th^ 2015. Five members of the rating group and the steering committee were present. The two items, on which there was an absence of consensus (baseline item 14 and cycle 2 item 3), were discussed. Baseline item 14 was finally judged as “appropriate” with a relative consensus and cycle 2 item 3 as appropriate with a strong consensus (Table 5).

## Discussion

This article reports findings from a modified Delphi study carried out to obtain a qualitative assessment of conformity to SPC criteria for cabazitaxel prescription in France. To our knowledge this is the first report of the use of the Delphi method for the prescription of an anticancer agent.

Our modified Delphi study involved two independent panels of experts in onco-urology. A first panel was composed of uro-oncology experts selected within the French national GETUG’s group. Of the 40 solicited experts 18 participated to the study as members of the rating committee. These experts belong to both cancer centres (11 members) and general hospitals (7 members) and were representative of all areas of France. The second independent panel was a European panel of experts who belonged to the genito-urinary group of oncologists from the European Organization for Research and Treatment of Cancer (EORTC). Of the 57 solicited experts, 16 accepted to participate to the study but only 15 experts provided answers to the second round questionnaire. All the European experts belonged to academic centres. The number of experts for each rating committee was similar to other studies developing prescription indicators in other fields than oncology such as paediatrics or general health care [8, 9]. The size of our panels is also in accordance with the published recommendations relative to the optimal size of a panel used for a Delphi method, i.e. 9–15 professional members [6, 7]. The limitation of our study is due to the nature of the rating panels that were restricted to oncologists specialized in the treatment of prostate cancer, which is inherent to the fact that cabazitaxel is only approved in that indication so far. This could eventually represent a bias in the analyses of our results as it precludes the participation of other oncology specialists with different backgrounds affecting decision-making process.

The results of our study show that among 26 items 15 were judged similarly by the two independent panels of experts including 11 items with strong consensus and 4 items with weak consensus (Table 5). Eight items were differently judged as weak or strong consensus items, depending on the panel. These items (baseline item 1, 4, 8, 9; cycle 2 item 1, 2, 3, 5) are mainly dealing with biological tests before treatment, with European oncologists being more stringent regarding white blood cells counts but less stringent regarding previous treatment by docetaxel and intervals between cycles.

Interestingly, the two items that were judged not appropriate by the French panel of experts are both related to the concomitant daily prescription of corticoids (prednisone or prednisolone) throughout the treatment. This indication may reflect the lack of studies that officially establish the benefits of prescribing corticoids during chemotherapy treatments. This item was also discussed during the conference call with the European panel and the experts preferred to judge this item with weak consensus in order to strictly follow the prescription guidelines though there was no rational to use it in combination.

For both rating panels, the items which were judged appropriate but with a lower consensus include AST, ALT and bilirubin levels. As precautionary measure and because cabazitaxel is metabolized by the liver, the drug should not be given to patients with hepatic impairment (levels of AST and/or ALT<1.5 ULN and bilirubin<1 ULN). However, it was known that in case of liver metastases chemotherapy can improve liver function; therefore, chemotherapy may be suitable even when aminotransferases and bilirubin levels are over the threshold. In the case of clinical trials eligibility, when liver metastases and impaired liver function are present, higher values of AST, ALT and bilirubin are accepted. Hence, depending on the patient’s situation, strict adherence to all SPC criteria might be judged not essential or restrictive by the clinician. The role of the practitioner in the evaluation of the patient is a fundamental aspect of treatment prescription and to the conformity to SPC indications, especially in the case of cytotoxic chemotherapies. Subjective judgment of the clinicians is often more important than strict adherence to some SPC indications, and gathering of experts’ opinion may somehow help to formalize such judgments.

Delphi consensus method can be used in several contexts and across several disciplines as a method to seek expert opinion in an iterative and structured manner. Indeed, it has been largely employed in the oncology field as means to define management criteria of certain types of cancers by the American College of Radiology [10–12] or for the assessment of time-to-event endpoint for cancer trials [13] or prostate cancer staging and surveillance [14], definition of risk [15] and therapy [16–18].

The two main features of this method are (i) anonymity, allowing opinions to be expressed free from the peer-group pressure, and (ii) iterative feedback, allowing participants to adjust their initial rating based on the feedback of the group rating. For our study, each member of the rating group received the two questionnaires by email and could fill them out privately; qualitative and quantitative feedbacks were also reported to the rating panel together with the second questionnaire. During the in-person meeting, only conclusions were formulated from the anonymously acquired data and no new input was given. It is also important to note that the European panel discussed the results of the second round without knowing the final results of the French panel.

In the frame of observatory studies it is often difficult to find concordance when numerous criteria need to be taken into account. It is therefore necessary to ponder each criterion in order to allow their qualitative assessment. Such assessment can entail a better and more controlled level of inclusion of patients in observatory studies as well as other clinical trials.

## Conclusion

Our study provides important evidence about the value of consensus methods to define primary objectives in observatory studies. Using such a methodology, we could objectively select criteria that could be used to evaluate conformity of cabazitaxel prescription in the context of observatory studies as well as in further clinical trials using this new taxane. Our results show that 11 items are of prime interest to ensure the quality of prescription of cabazitaxel. They further highlight a need for “conformity” standards to assist practitioners for the prescription of recently approved chemotherapies.

## Supporting Information

S1 Table Analysis of the first round of rating according to RAND methodology (7). (DOCX)

S2 Table Analysis of the second round of rating according to RAND methodology (7). (DOCX)
