# Supplementary material for: Formal Consensus Method to Evaluate the Conformity of Prescription of a Recently Approved Chemotherapy Treatment in an Observatory Study
Source: PLoS One. 2015 Apr 2;10(4):e0123035. doi: 10.1371/journal.pone.0123035 (PMC4383448; doi:10.1371/journal.pone.0123035)
Supplement: S1 Table — (DOCX) [file pone.0123035.s001.docx]

**S1 Table**: Analysis of the first round of rating according to RAND methodology.

| **Item defined as** | **Degree of agreement** |  | |
| --- | --- | --- | --- |
|  |  | **Median** | **Distribution of responses** |
| **Appropriate** | Strong Consensus | ≥7 | [7-9] |
|  | Relative consensus | ≥7 | [5-9] |
| **Inappropriate** | Strong Consensus | ≤3 | [1-3] |
|  | Relative Consensus | ≤3.5 | [1-5] |
| **Uncertain** | Indecision | 4< Median> 6.5 | [1-9] |
|  | No Consensus | All other options | |
